# Suppression of miR-22, a tumor suppressor in cervical cancer, by human papillomavirus 16 E6 via a p53/miR-22/HDAC6 pathway

**DOI:** 10.1371/journal.pone.0206644

**Published:** 2018-10-31

**Authors:** Weerayut Wongjampa, Tipaya Ekalaksananan, Peechanika Chopjitt, Jureeporn Chuerduangphui, Pilaiwan Kleebkaow, Natcha Patarapadungkit, Chamsai Pientong

**Affiliations:** 1 Department of Microbiology, Faculty of Medicine, Khon Kaen University, Khon Kaen, Thailand; 2 HPV & EBV and Carcinogenesis Research Group, Khon Kaen University, Khon Kaen, Thailand; 3 Faculty of Public Health, Kasetsart University Chalermphrakiat, Sakon Nakhon Campus, Sakon Nakhon, Thailand; 4 Department of Obstetrics and Gynecology, Faculty of Medicine, Khon Kaen University, Khon Kaen, Thailand; 5 Department of Pathology, Faculty of Medicine, Khon Kaen University, Khon Kaen, Thailand; University of South Alabama Mitchell Cancer Institute, UNITED STATES

## Abstract

MicroRNAs (miRNAs) are small non-coding RNAs that function to down-regulate gene expression involving in various cellular processes related to carcinogenesis. Recently, miR-22 was identified as a tumor-suppressing miRNA in many human cancers. However, the regulatory mechanism and the specific function of this miRNA in cervical cancer remain unclear. In the present study, we carried out gene transfection, western blot and quantitative RT-PCR to explore the regulatory mechanism and the functional role of miR-22 in cervical cancer. We verified that miR-22 was down-regulated in cervical cancer tissues and cervical cancer cell lines relative to matched non-tumor tissues and normal human cervical keratinocyte line (HCK1T). By contrast, histone deacetylase 6 (HDAC6) was inversely correlated with miR-22 in both cervical tissues and cancer cell lines. Mechanically, HDAC6 was down-regulated by miR-22 at the post-transcriptional level, via a specific target site within the 3’UTR, identified by a luciferase reporter assay. Moreover, we also showed that the correlation between miR-22 and HDAC6 expression was regulated by an E6/p53 pathway in HCK1Ts expressing HPV16 E6. For functional study, an ectopic expression of miR-22 could inhibit cell proliferation and migration, and could induce apoptosis of cervical cancer cell lines. These findings demonstrated that miR-22 was down-regulated in cervical cancer and inversely collated with its downstream target HDAC6. MiR-22 acts as tumor suppressor by inhibiting proliferation and migration, and by inducing apoptosis of cervical cancer cell lines by targeting the 3’UTR of HDAC6. This newly identified E6/p53/miR-22/HDAC6 regulatory network might be a candidate therapeutic target for cervical cancer.

## Introduction

Cervical cancer is one of the most malignant tumors. There were an estimated 527,600 new cervical cancer cases and 265,700 deaths worldwide in 2012 [1]. Surgery, radiotherapy and chemotherapy are the major methods in the treatment of cervical cancer [2]. However, effective targeted therapeutic drugs are not yet available. Therefore, the search for the novel therapeutic targets and the development of specific drugs for cervical cancer treatment is very important. It is recognized that cervical cancer is almost always caused by persistent infection with high-risk human papillomaviruses (HR-HPVs). HPV has been implicated in 99.7% of cervical cancer cases worldwide and 70.1% of cases of cervical cancer are attributed to HR-HPV types 16 and 18 [3, 4]. These viruses encode two oncoproteins, E6 and E7, which are consistently expressed in human cervical cancer cells and possess oncogenic activities including the ability to transform and immortalize keratinocytes, the host cells of HPV [5, 6].

At present, the pathogenesis mechanisms of cervical cancer are not entirely clear. Inactivation of tumor suppressor genes and activation of oncogenes play a significant role in carcinogenesis. With deeper understanding of tumor biology in recent years, increasing evidence has shown that epigenetic alteration plays an important role. Epigenetic changes include DNA methylation, chromatin remodeling, histone modification, and microRNA (miRNA) regulation [7].

miRNAs are a class of small noncoding RNAs that down-regulate the translation of target protein-coding mRNAs at the 3’ untranslated region (UTR). Accumulating evidence has shown that miRNAs are involved in multiple processes in cancer development and progression [8, 9]. Recently, miR-22 has been identified as a tumor-suppressing miRNA and its expression was decreased in a variety of human neoplasms, including hepatocellular carcinoma [10], colorectal cancer [11], gastric cancer [12], lung cancer [13], breast cancer [14], cervical cancer and also in raft cultures of human foreskin keratinocytes (HFKs) transduced with HPV18 E6 [9]. However, the regulatory mechanism and the specific function of this miRNA in cervical cancer are still unclear. In one report, miR-22 was identified as a direct transcriptional target of p53 [15]. In addition, a functional study of miR-22 in human adipose tissue-derived mesenchymal stem cells identified histone deacetylase 6 (HDAC6) as a direct downstream target of this miRNA and associated with osteogenic and adipogenic differentiation [16]. In cervical cancer cells, p53 protein was suppressed by E6 oncoprotein, the E6-E6AP complex binds to p53 and stimulates its degradation [17]. Therefore, we hypothesize that the altered expression of miR-22 and its downstream target, HDAC6, in cervical cancer may be controlled by the E6-p53 pathway and involved in cervical cancer development and progression.

In the present study, we determined the expression levels of miR-22 and its downstream target, HDAC6, in cervical cancer cells and tissue samples. Then the ability of HPV16 E6 to down-regulate miR-22 and the effects of miR-22 on post-transcriptional repression of HDAC6 were investigated. In addition, the biological functions of miR-22 in proliferation, migration and apoptosis of cervical cancer cells were determined to better elucidate mechanisms by which the E6/miR-22 pathway might influence cervical carcinogenesis. These findings may provide an insight into the interaction network of HPV oncogenes, miRNA and target genes, which might suggest a potential target in application of cervical cancer therapy.

## Materials and methods

### Clinical specimens

This is a retrospective study which uses leftover specimens from the previous projects. Cervical samples, including fresh tissues and formalin-fixed, paraffin-embedded (FFPE) tissues, were obtained from women who had undergone routine cervical cancer investigation by Papanicolaou (Pap) smear testing in combination with colposcopy at Srinagarind Hospital and Khon Kaen Hospital, Khon Kaen, Thailand. All had provided their written informed consent to participate in previous projects approved by the Human Research Ethics Committee of Khon Kaen University (no. HE581455). All women included in the study were examined by gynecologist first: gynecological and cytological examination was performed for all women. Patients with abnormal cytology were enrolled in this study. Each patient underwent a colposcopy-directed biopsy. FFPE and H&E staining were performed for each specimen, which was examined by a pathologist to verify the quality of the sample. None of the patients recruited in this study had undergone preoperative chemotherapy or radiotherapy. Normal cervical tissues without HPV infection were collected from patients who underwent hysterectomy for nonmalignant conditions. The specimens were grouped according to the histological diagnosis reviewed by the pathologist. Seventy-two HR-HPV-positive fresh tissues were histologically classified into three groups: low-grade squamous intraepithelial lesion; LSIL (n = 22), high-grade squamous intraepithelial lesion; HSIL (n = 20), squamous cell carcinoma; SCC (n = 30). An additional 30 samples were HPV-negative tissues with no squamous intraepithelial lesions; NoSIL (n = 30), used as controls in some experiments. Twenty-five HR-HPV-positive FFPE tissues were histologically classified into two groups: HSIL (n = 14) and SCC (n = 11). HPV infection status of the cervical tissue biopsies was determined by PCR and HPV reverse line blot hybridization. Characteristics of patients with cervical cancer were shown in supporting information: Table A in S1 File.

### Cell cultures

The human cervical cancer cell lines C33A, HeLa, SiHa and CaSki were cultured in Dulbecco’s modified Eagle’s medium (DMEM) (Gibco-Life Technologies, Grand Island, NY, USA) supplemented with 10% fetal bovine serum (FBS) (Gibco-Life Technologies) and antibiotics (Gibco-Life Technologies). The immortalized human cervical keratinocyte line expressing HPV16 E6 (HCK1T, Tet-On system), kindly provided by Prof. Tohru Kiyono (National Cancer Center Research Institute, Japan), was cultured in keratinocyte serum-free medium (KSFM) supplemented with 5 ng/ml epidermal growth factor (EGF) and 50 μg/ml bovine pituitary extract (BPE). The cell line was induced by doxycycline treatment (Gibco-Life Technologies, Grand Island, NY, USA) to start production of HPV16 E6. The human embryonic kidney cell line 293FT (Invitrogen, Carlsbad, CA, USA) was maintained in DMEM supplemented with 10% FBS and antibiotics. All cells were maintained in an incubator with an atmosphere of 5% CO_2_ at 37°C.

### Protein and RNA detection in HCK1T expressing HPV16 E6

HCK1T cells (2.5 x 10^5^) were seeded into 100 x 20 mm tissue culture dishes (SPL Life Sciences, Seoul, Korea) and allowed to attach to the plate surface for 24 h. In the Tet-On system, HPV16 E6 expression was induced by adding 1 μg/ml doxycycline (Dox^+^). At three time points after addition of doxycycline (24, 48 and 72 h), cells were trypsinized and washed for protein isolation with radio immunoprecipitation assay (RIPA) buffer (25 mM Tris–HCl pH 7.6, 150 mM NaCl, 1% sodium deoxycholate, 0.1% SDS). P53, HDAC6 and actin levels in HCK1T cells expressing HPV16 E6 were determined by western blot and compared with controls not expressing HPV16 E6 (Dox^-^). RNA was extracted and reverse-transcribed into cDNA to determine expression of HPV16 E6, GAPDH, miR-22 and U44.

### Plasmid construction and transient transfections

For the expression of miR-22, miRNA stem loop sequence was downloaded from Sanger Institute’s miRNA database (http://microrna.sanger.ac.uk/sequences/) (Supporting information: Table B in S1 File) and blasted the search miRNA stem loop sequence in the NCBI (http://blast.ncbi.nlm.nih.gov/Blast.cgi). Native flank sequences (100 bases) were added both upstream and downstream of the miRNA stem loop. PCR primers incorporating restriction enzyme cleavage sites were designed (Supporting information: Table C in S1 File). The *Homo sapiens* miR-22 precursor was amplified by PCR (Supporting information: Table D in S1 File) and cloned into pGEM-T cloning vector (Promega, Madison, WI, USA) then sub-cloned into a pIRES-2-EGFP expression vector (BD Biosciences Clontech, Palo Alto, CA) and named miR-22/pIRES-2-EGFP. The constructed vector was confirmed by DNA sequencing. For transient transfection with miR-22 expression plasmid, 5x10^6^ cells/sample were transfected with 200 ng of miR-22/pIRES-2-EGFP or mock/pIRES-2-EGFP using Lipofectamine 2000 transfection reagent (Invitrogen, Carlsbad, CA, USA).

### Total RNA isolation and quantitative RT-PCR

Total RNA was isolated from cells and fresh cervical tissues using Trizol reagent (Invitrogen, Carlsbad, CA, USA). For RNA extraction from FFPE tissues, each paraffin-embedded cervical tissue block was trimmed and cut for a 10 μm-thick section. Six sections were mounted on slides under RNase-free conditions and air dried for about 2 h at room temperature. Then, they were deparaffinized with xylene and stained with hematoxylin and eosin stain. After staining, laser capture microdissection (LCM) was used to sample from selected tumor regions and normal regions. The procedures were as follows: a tissue fraction step by selection of 20–100 x 10^3^ μm^2^ (4–6 areas) of normal epithelium or cervical epithelium/cancer cells isolated into separate cups using the PALM Carl ZeissMicroImmaging Laser Capture Microdissection system (Carl Zeiss Microscopy, Jena, Germany). Total RNA was isolated using High Pure RNA Paraffin Kit (Roche Applied Science, Penzberg, Germany) and quantified using the NanoDrop^TM^ 2000/2000c spectrophotometer (Thermo-Fisher, Massachusetts, USA). Total RNA (1 μg) was reverse-transcribed into cDNA using a Super Script III First-strand Synthesis Kit (Invitrogen, Carlsbad, CA, USA). Before reverse transcription, all RNA samples were routinely treated with DNase I (Promega, Wisconsin, USA) to remove any contaminating DNA. Quantitative RT-PCR was performed with gene specific primers for HPV E6, p53, HDAC6 and the house-keeping gene GAPDH (Sigma-Aldrich, MO, USA) using the SYBR Green PCR Master Mix (Bio-Rad, Hercules, CA, USA). For miR-22 detection, total RNA (1 μg) was reverse-transcribed into cDNA using a MystiCq microRNA cDNA Synthesis Mix (Sigma-Aldrich, MO, USA). Quantitative RT-PCR for miR-22 was performed using MystiCq microRNA SYBR Green qPCR Ready Mix (Sigma-Aldrich, MO, USA). U44 was used as an internal control. Threshold cycle numbers (CT) were determined with LightCycler 480 SYBR Green I Master Real-time PCR (Roche Applied Science, Penzberg, Germany). Relative quantification of the mRNA and miRNA expression was calculated with the 2^-ΔΔCT^ method. The primer sequences and RT-PCR conditions were shown in Supporting information: Tables C and E in S1 File.

### Western blot analysis

The cells were washed twice with cold PBS and total cellular protein was extracted using RIPA buffer. The isolated proteins were quantified using the Bradford assay (Bio-Rad, Richmond, CA, USA). Total protein was separated by SDS-polyacrylamide gel electrophoresis (SDS-PAGE) and transferred to nitrocellulose membranes by semidry blotting (Bio-Rad, CA, USA). The membrane was blocked with 5% skim milk and incubated with antibodies against p53 (Santa Cruz Biotech, CA, USA; 1:1000 dilution), HDAC6 (Cell Signaling Technology; 1:1000 dilution) or β-actin (Sigma-Aldrich, MO, USA; 1:2000 dilution) and then incubated with peroxidase-conjugated secondary antibody (Santa Cruz Biotech, CA, USA; 1:10000 dilution). Finally, the blots were washed and the signals were visualized with enhanced chemiluminescence reagents (GE Healthcare, Uppsala, Sweden). The intensity of target protein bands was standardized to the intensity of the β-actin bands. The intensity of protein bands was measured using Image J 1.49v software (National Institutes of Health, Bethesda, MD, USA).

### Target prediction and luciferase activity assay

Prediction of miRNA target relied on the databases TargetScan (WWW.targetscan.org/) and miRanda (WWW.microrna.org/). The full length 3’UTR of HDAC6 was amplified by PCR from genomic DNA using specific primers (Supporting information: Tables C and D in S1 File) and cloned into pGEM-T cloning vector (Promega, Madison, WI, USA) then sub-cloned into pGL3 control vector (Promega, Madison, WI, USA). The constructed vector was confirmed by DNA sequencing. Co-transfection of HDAC6 3’UTR/pGL3 Control and miR-22/pIRES-2-EGFP expression vectors was performed using Lipofectamine 2000 transfection reagent (Invitrogen, Carlsbad, CA, USA). After 48 h, luciferase activity was measured using a dual luciferase reporter assay system according to the manufacturer’s protocol (Promega, Madison, WI, USA).

### Cell proliferation assays

C33A (4x10^4^ cells/well) and HeLa (4x10^4^ cells/well) cells were cultured separately in 96-well plates overnight, then transfected with pIRES-2-EGFP or miR-22/pIRES-2-EGFP. At 24, 48 and 72 h after transfection, cell growth activities were examined using the 3-(4,5-dimethyl-2-thiazolyl)-2,5-diphenyl-2H-tetrazolium bromide (MTT) assay kit (Sigma-Aldrich, MO, USA). The absorbance of samples was recorded at 570 nm using a microplate spectrophotometer (TECAN, Salzburg, Austria).

### Wound healing assay

C33A (1x10^5^ cells/well) and HeLa (4x10^5^ cells/well) cells were cultured separately in 24-well plates overnight, then transfected with pIRES-2-EGFP or miR-22/pIRES-2-EGFP. When the culture had reached nearly 90% confluence, the cell layer was scratched with a sterile plastic tip and then washed with culture medium twice and cultured again for 24 or 48 h with serum-reduced medium (DMEM, with 1% FBS). At different time points, photographic images of the wound size were acquired under a microscope and the data were summarized.

### Cell apoptosis analysis

C33A (1 x 10^5^ cells/well) and HeLa (4 x 10^5^ cells/well) cells were cultured separately in 6-well plates overnight, then transfected with pIRES-2-EGFP or miR-22/pIRES-2-EGFP. At 48 h following transfection, a flow cytometer was used to detect cell apoptosis. Briefly, apoptotic cells were stained using annexin-V-FITC and propidium iodide (PI) (Invitrogen, Carlsbad, CA, USA) according to the manufacturer’s protocol. After incubation, cells were analyzed using flow cytometry (Becton Dickinson FACSCanto II, USA). Triplicate independent experiments were performed.

### Statistical analysis

The data are presented as the mean ± SEM (standard error of the mean) and were compared using Student’s t-test or oneway ANOVA test, as appropriate in the Statistical Program for Social Sciences 13.0 software (SPSS Inc., Chicago, IL, USA), and results were considered statistically significant at *P* < 0.05. All graphs were drafted using GraphPad Prism version 5.00.286 (GraphPad Prism, San Diego, California, USA).

## Results

### The expression of miR-22 was down-regulated in HSIL and SCC, as well as in cervical cancer-derived cell lines

**Fig 1:**
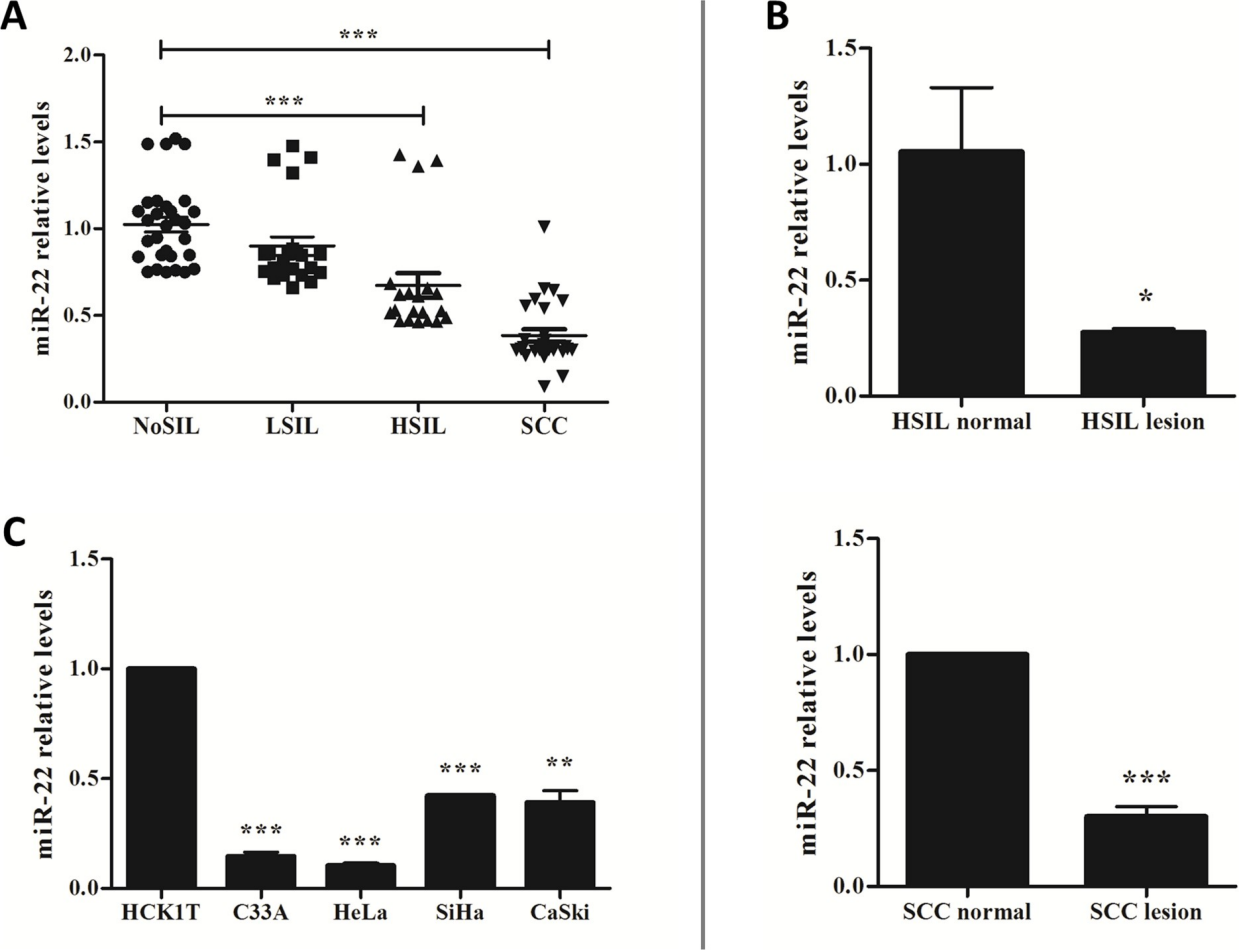
miR-22 was down-regulated in cervical cancer tissues and cell lines. **A.** The relative levels of miR-22 in the different grades of fresh-frozen cervical tissues. **B.** Comparison of miR-22 expression levels between lesion tissues and adjacent normal tissues in HSIL and SCC FFPE samples. **C.** The relative levels of miR-22 in four cervical cancer cell lines and one immortalized human cervical keratinocyte line (HCK1T). The levels of miR-22 were measured by qRT-PCR and normalized with U44 small nuclear RNA. *P < 0.05, **P < 0.01, ***P < 0.001.

The expression of miR-22 was examined by quantitative real-time reverse-transcriptase PCR (qRT-PCR) in fresh frozen cervical tissues including NoSIL, LSIL, HSIL and SCC, as well as in laser capture microdissected FFPE cervical samples including HSIL and SCC. As shown in Fig 1A and Fig A in S1 File, we found that miR-22 was down-regulated in HSIL (0.62 ± 0.14) and SCC (0.39 ± 0.11) samples compared with NoSIL (1.02 ± 0.12) samples (*P* < 0.001) but there was no difference between LSIL (0.90 ± 0.13) and NoSIL. In addition, the expression of this miRNA was also significantly lower in tumor regions compared with normal regions in both HSIL and SCC of FFPE samples (Fig 1B and Fig A in S1 File). Similarly, we also found that expression of miR-22 was much less in four cervical cancer cell lines than in an immortalized human cervical keratinocyte line (HCK1T) (Fig 1C). Our findings showed that the tendency of reduced miR-22 expression was consistent with that of cervical lesion progression, indicating that miR-22 might play an important role in cervical cancer progression.

### The expression of HDAC6 was up-regulated in HSIL and SCC, as well as cervical cancer-derived cell lines

**Fig 2:**
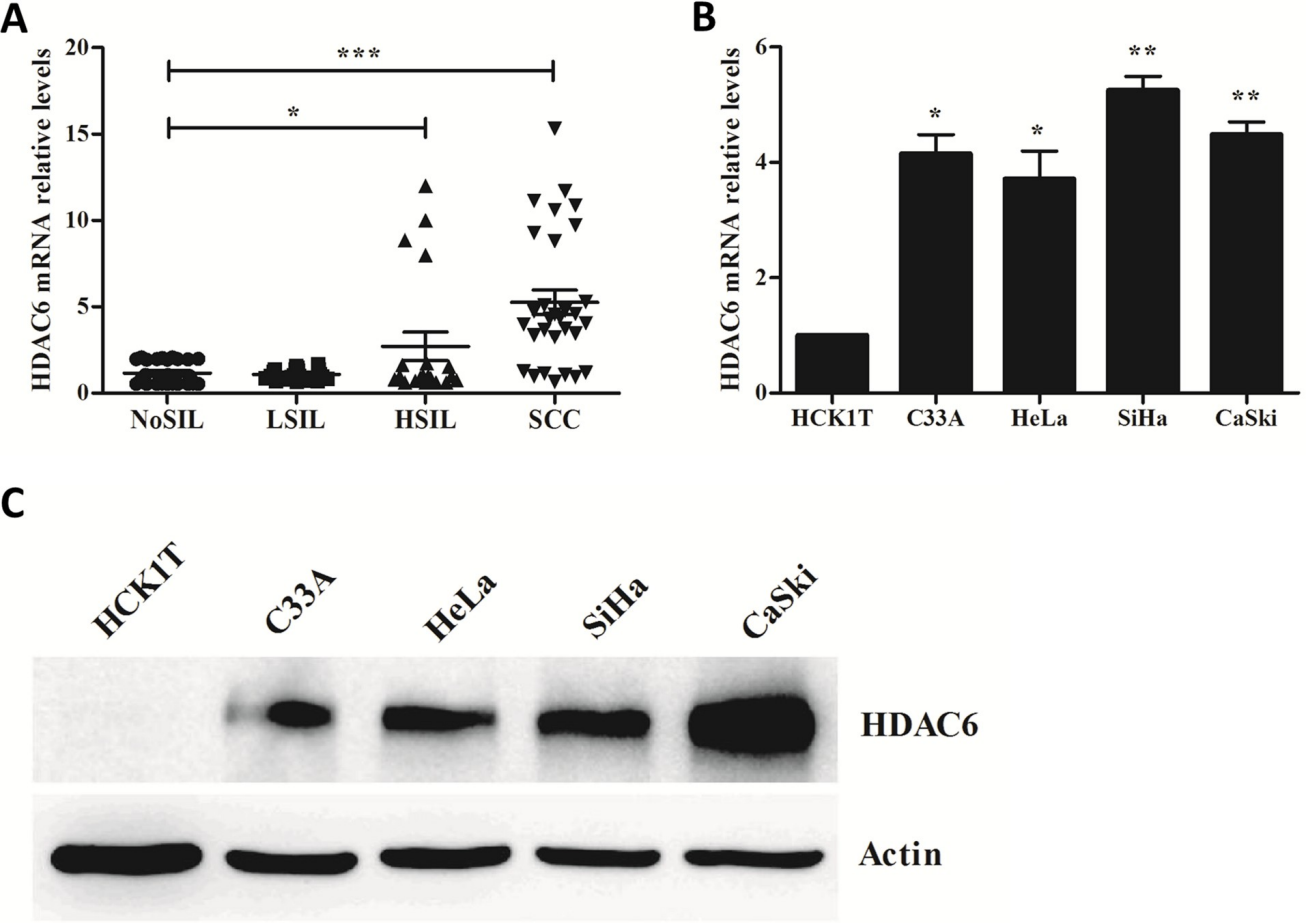
HDAC6 was up-regulated in cervical cancer tissues and cell lines. **A.** The relative levels of HDAC6 in the different grades of fresh-frozen cervical tissues. **B.** The relative levels of HDAC6 in four cervical cancer cell lines and one immortalized human cervical keratinocyte line (HCK1T). The levels of HDAC6 were measured by qRT-PCR and normalized with GAPDH. **C.** The expression levels of HDAC6 protein in four cervical cancer cell lines and one immortalized human cervical keratinocyte line (HCK1T) were measured by western blotting. *P < 0.05, **P < 0.01, ***P < 0.001.

The expression of HDAC6 was examined by qRT-PCR in fresh frozen cervical tissues including NoSIL, LSIL, HSIL and SCC. As shown in Fig 2A and Fig B in S1 File, we found that HDAC6 mRNA was significantly up-regulated in HSIL (2.72 ± 1.83) and SCC (5.26 ± 1.95) samples compared with NoSIL (1.15 ± 0.32) samples but there was no difference between LSIL (1.09 ± 0.16) and NoSIL. Similarly, we also found that expression of HDAC6 was higher in all of four cervical cancer cell lines than in an immortalized human cervical keratinocyte line (HCK1T) (Fig 2B and 2C). Increased HDAC6 expression was consistent with cervical lesion progression, indicating that HDAC6 might play an important role in cervical cancer progression.

### miR-22 targets the 3’UTR of the HDAC6 and down-regulates its expression

**Fig 3:**
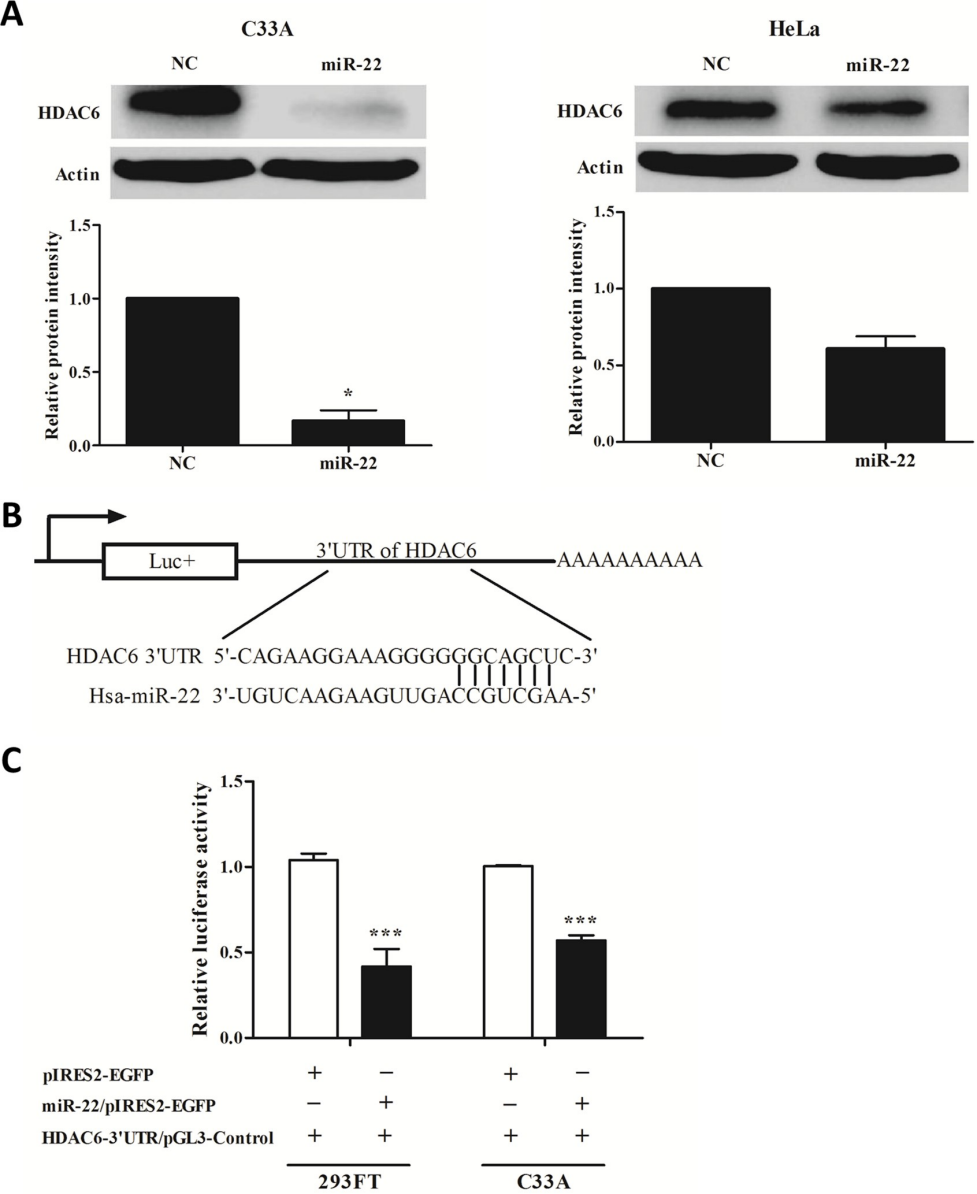
HDAC6 is a target of miR-22. **A.** miR-22 inhibited the expression of HDAC6 in cervical cancer cells. **B.** Predicted duplex formation between human HDAC6 and miR-22. **C.** The relative luciferase activities in 293FT and C33A cells were determined after the HDAC6 3’UTR plasmid was co-transfected with miR-22. Error bars indicate the standard error of the mean (SEM) of triplicated independent experiments. *P < 0.05, ***P < 0.001.

miRNAs are known to suppress hundreds of mRNA targets, resulting in global changes in the cellular phenotype of cells [8]. First, we made an effort to identify potential targets for miR-22 using software prediction approaches. We used a consensus approach with two widely used types of software (miRanda and TargetScan) to perform the target prediction. We found the gene HDAC6 as the putative target gene for miR-22. HDAC6 is a key regulator of many signaling pathways that are linked to cancer [18]. To confirm further that HDAC6 is a target gene for miR-22, western blot analysis was used to detect the expression of HDAC6 in the presence or absence of miR-22 in C33A and HeLa cells. HDAC6 was down-regulated after overexpression of miR-22 (Fig 3A). Next, to verify whether miR-22 directly targeted HDAC6, luciferase reporter assays were conducted. We constructed a HDAC6-3’UTR/pGL3 control vector (Fig 3B). Co-transfection of 293FT with HDAC6-3’UTR/pGL3 control vector and miR-22/pIRES-2-EGFP caused a 60% decrease in the luciferase activity compared with the negative control (*P* < 0.001). A similar effect was also found in C33A cells (40% decrease compared with the negative control, *P* < 0.001) (Fig 3C). All these results indicated that miR-22 exerts inhibitory effects on HDAC6 expression via interaction with the 3’UTR of HDAC6.

### The expression of miR-22 and HDAC6 was regulated by E6/p53 pathway

**Fig 4:**
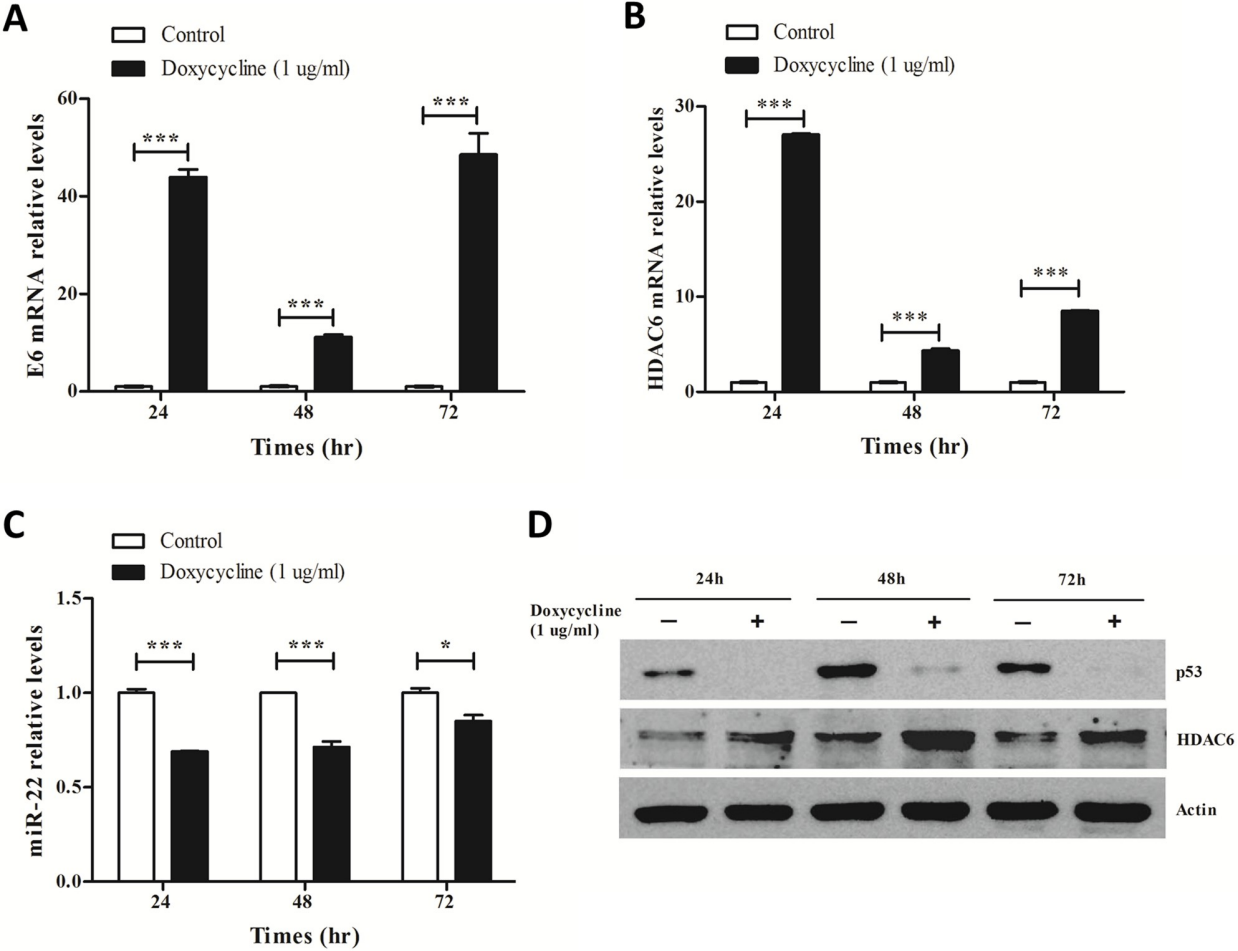
E6, p53, miR-22 and HDAC6 expression levels in HCK1T cells expressing HPV16 E6. 1x10^6^ cells were seeded in 60-mm tissue culture dishes and maintained in growth medium with/without 1 μg/ml doxycycline. Expression levels of E6 mRNA (**A**), HDAC6 mRNA (**B**) and miR-22 (**C**) at different time points were examined by qRT-PCR. **D.** p53 and HDAC6 protein levels were analyzed by western blot. Error bars indicate the standard error of the mean (SEM) of triplicated independent experiments. *P < 0.05, ***P < 0.001.

Considering that miR-22 was identified as a direct transcriptional target of p53 [15] and p53 protein was suppressed by E6 oncoprotein, it is likely that miR-22 expression may be dysregulated in cells expressing HPV E6. We therefore examined the expression profile of miR-22 and its downstream target HDAC6 in HCK1T cells stimulated to express HPV16 E6 by doxycycline treatment (Tet-On system). Levels of HPV16 E6, HDAC6 mRNA and miR-22 were measured using qRT-PCR. HDAC6 and p53 protein levels were analyzed using western blot. We found that E6 mRNA was up-regulated after doxycycline treatment compared with untreated cells (Fig 4A). Levels of HDAC6 mRNA and protein increased at each time point (24–72 h) in cells expressing HPV16 E6 compared with controls as shown in Fig 4B and 4D. Correspondingly, miR-22 and p53 protein levels decreased at each time point (Fig 4C and 4D). These results demonstrate that the involvement of p53 in the posttranscriptional regulation of HDAC6 expression occurs via miR-22. In HCK1T cells expressing HPV16 E6, p53 protein was suppressed by E6 oncoprotein resulting in diminished miR-22 transcription, which in turn leads to enhanced HDAC6 expression and may be involved in the development of cervical cancer.

### Overexpression of miR-22 inhibits proliferation and migration of cervical cancer cells

**Fig 5:**
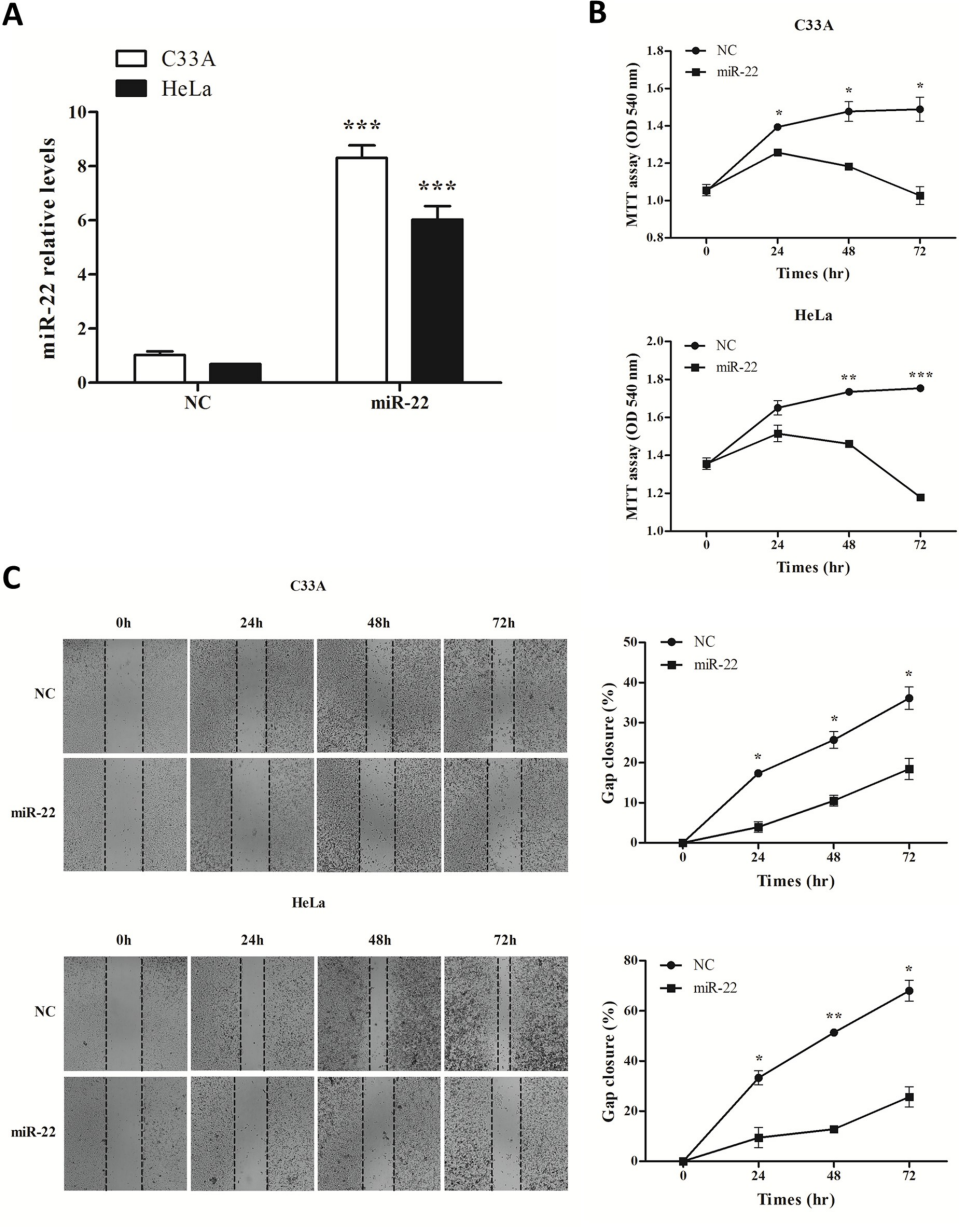
Overexpression of miR-22 inhibited proliferation and migration of cervical cancer cell lines. **A.** Transfection of miR-22 expression plasmid to C33A and HeLa cells increased the expression of miR-22 detected by real-time quantitative RT-PCR. **B.** Cell proliferation of these cells transfected as in (A) was measured at the indicated time periods using the MTT assay. **C.** Overexpression of miR-22 led to a slower closing of scratch wounds, compared with negative control transfected cells. Error bars indicate the standard error of the mean (SEM) of triplicated independent experiments. *P < 0.05, **P < 0.01, ***P < 0.001.

C33A and HeLa cells were transfected with the miR-22 expression vector or pIRES-2-EGFP control vector. As expected, transfection of miR-22 expression plasmid into C33A and HeLa cells resulted in increase in miR-22 expression compared with negative control (NC) transfected cells (Fig 5A).

To examine the role of miR-22 in the proliferation and migration of cervical cancer cells, we preformed MTT cell proliferation and wound-healing assays with transfected miR-22 in C33A and HeLa cells. Our results showed that restoration of miR-22 expression suppressed proliferation in both cervical cancer cell lines (Fig 5B). Next, the wound-healing assay showed that C33A and HeLa cell lines over-expressing miR-22 exhibited a slower closing of scratch wounds, compared with the negative controls (Fig 5C). Our results indicate that miR-22 worked as a tumor-suppressing miRNA, reducing proliferation and migration of cervical cancer cells.

### Over expression of miR-22 induce cervical cancer cells apoptosis

**Fig 6:**
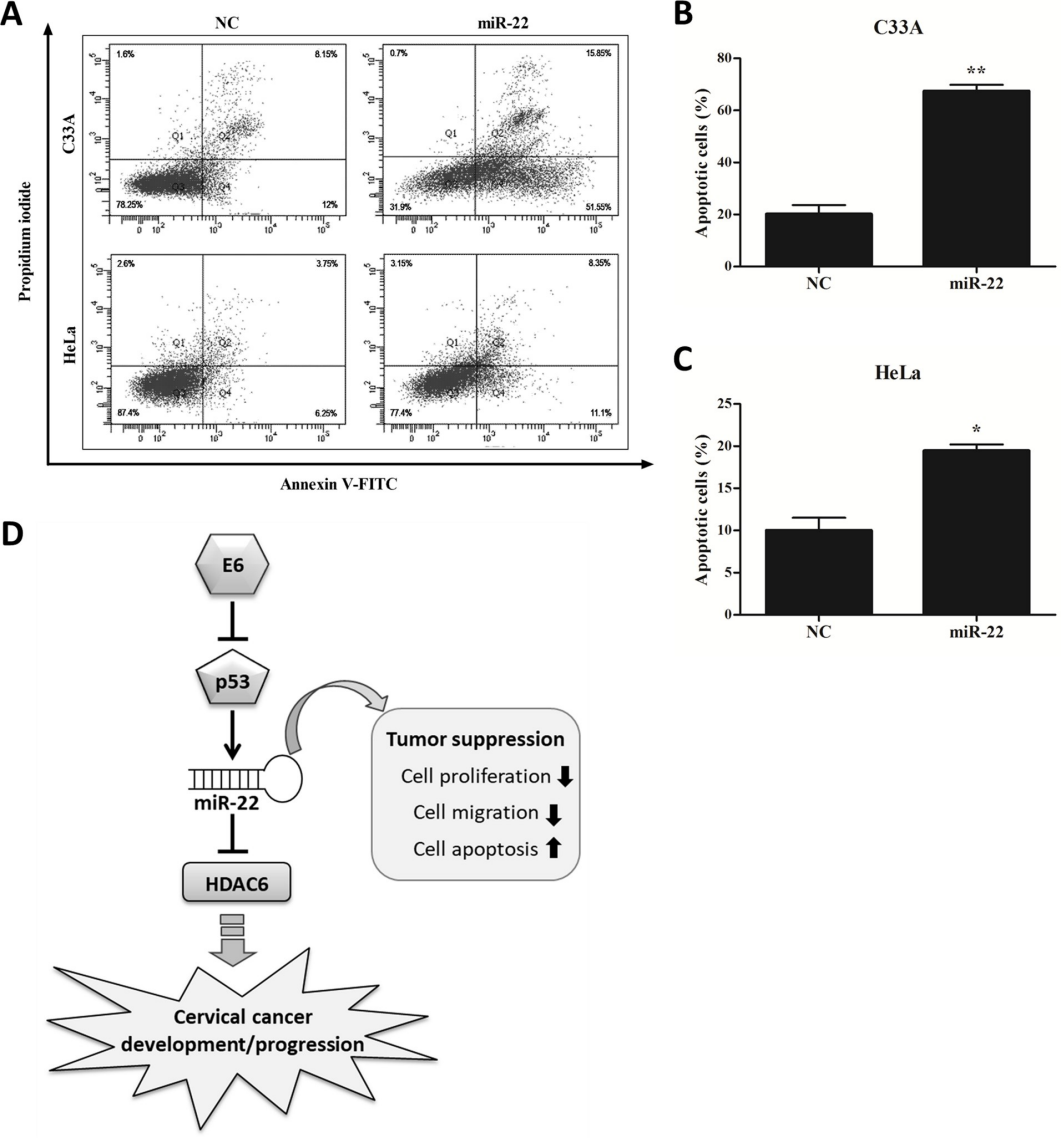
miR-22 induced cervical cancer cells apoptosis. Flow cytometric assay showed the increased fractions of apoptotic cells in C33A and HeLa cell lines transfected with miR-22 (**A**). Mean fraction of apoptosis in C33A (**B**) and HeLa (**C**) cells. The values represent apoptotic cells. Error bars indicate the standard error of the mean (SEM) of triplicated independent experiments. *P < 0.05, **P < 0.01. **D.** Hypothetical mechanism by which HPV E6 induces cervical cancer development/progression through the p53/miR-22/HDAC6 pathway. Modulation of tumor suppressor miR-22 and its down-stream target HDAC6 by the HPV E6/p53 pathway is involved in the development process of cervical cancer.

To figure out the effect of miR-22 on cervical cancer cell apoptosis, C33A and HeLa cells were transfected with miR-22 expression vector or empty vector and then subjected to a flow cytometric assay. MiR-22 overexpression significantly increased apoptosis in C33A (66.5 ± 2.4% vs. 21.7 ± 3.45%; *P* < 0.01; Fig 6A and 6B) and in Hela (18.95 ± 0.75% vs. 10.5 ± 1.5%; *P* < 0.05; Fig 6A and 6C), compared with their respective empty vector controls.

## Discussion

MiRNAs play an important role in cancer development and progression in many types of cancers [19–21]. Studies have focused on cancer-specific miRNAs and associated target genes to elucidate biological mechanisms [22, 23]. Previous reports suggested that miR-22 was down-regulated in various cancers including breast cancer [14], colon cancer [24], pancreatic cancer [25] and cervical cancer [26]. However, miR-22 is apparently up-regulated in prostate cancer, thus potentiating host oncogene activation [27]. These controversial results of miR-22 in cancer development may reflect the diverse roles of miR-22 in different types of cancer.

In this study, we demonstrated that miR-22 was down-regulated in cervical cancer tissues and cell lines. In addition, we also confirmed the role of miR-22 in cervical cancer via its ability to inhibit cell proliferation and migration, and to induce cell apoptosis. Corresponding with a previous report, miR-22 suppressed cell proliferation and invasion and migration of cervical cancer cells [26]. Taken together, our results suggest that miR-22 acts as a tumor suppressor and plays a role in cervical carcinogenesis.

Target genes of miR-22 so far reported include HDAC4, CDK6, SIRT1, SP1, HIF-1α and PTEN, which are involved in cancer progression [28]. In addition, the functional study of miR-22 in human adipose tissue-derived mesenchymal stem cells found that HDAC6 was a direct downstream target of this miRNA [16]. HDAC6 is usually recognized as a key regulator of many signaling pathways that are linked to cancer [29]. We used a public algorithm to find that HDAC6 was a putative target gene for miR-22. We found an inverse correlation of miR-22 and HDAC6 expression in cervical cancer cells and tissue samples. As expected, HDAC6 was negatively regulated by miR-22 at the posttranscriptional level, via a specific target site within the 3’UTR. This is the first report that HDAC6 is regulated by miRNA in cervical cancer. However, molecular mechanisms by which expression of miR-22 is reduced in cervical cancer remain to be elucidated.

Recent studies have discovered that host cell miRNAs are dysregulated in high-risk HPV-infected cervical cancer tissues and cells [30, 31]. Due to the known significant carcinogenesis roles of E6 and E7, researchers have attempted to find whether the differentially expressed miRNAs were regulated by E6 or E7 [32, 33]. In one previous study, miR-22 was identified as a direct transcriptional target of p53 [15]. In cervical cancer cells, p53 protein was suppressed by E6 oncoprotein [17]. Therefore, we hypothesize that the altered expression of miR-22 may be controlled by the E6/p53 pathway. In this study, induced expression of HPV16 E6 in HCK1T cells resulted in reduced production of p53 and its downstream target miR-22. The level of HDAC6 increased in line with E6 levels. This is the first demonstration of the mechanism of HPV16 E6 down-regulating miR-22 via the E6/p53/miR-22 pathway.

In summary, down-regulation of miR-22 was found in cervical cancer tissues and cell lines. This molecule acts as tumor suppressor by inhibiting proliferation and migration, and inducing apoptosis of cervical cancer cells. HDAC6 was identified as a direct downstream target of miR22: an inverse correlation of these two molecules was found. It seems that miR-22 dysregulation may impact on HDAC6 induction, possibly promoting cervical carcinogenesis (Fig 6D). Furthermore, we also demonstrated that miR-22 was regulated by the E6/p53/miR-22 pathway. This study is one step forward in understanding miR-22-dependent regulation, provides an insight into the interaction network of viral oncogenes, miR-22 and HDAC6, and might indicate a target in application for therapy of cervical cancer in the future.

## Supporting information

S1 File Containing Figures A and B and Tables A-E.
(DOCX)
