# Supplementary material for: Suppression of miR-22, a tumor suppressor in cervical cancer, by human papillomavirus 16 E6 via a p53/miR-22/HDAC6 pathway
Source: PLoS One. 2018 Oct 31;13(10):e0206644. doi: 10.1371/journal.pone.0206644 (PMC6209303; doi:10.1371/journal.pone.0206644)
Supplement: S1 File — Containing Figures A and B and Tables A-E. (DOCX) [file pone.0206644.s001.docx]

**Supporting information**

**
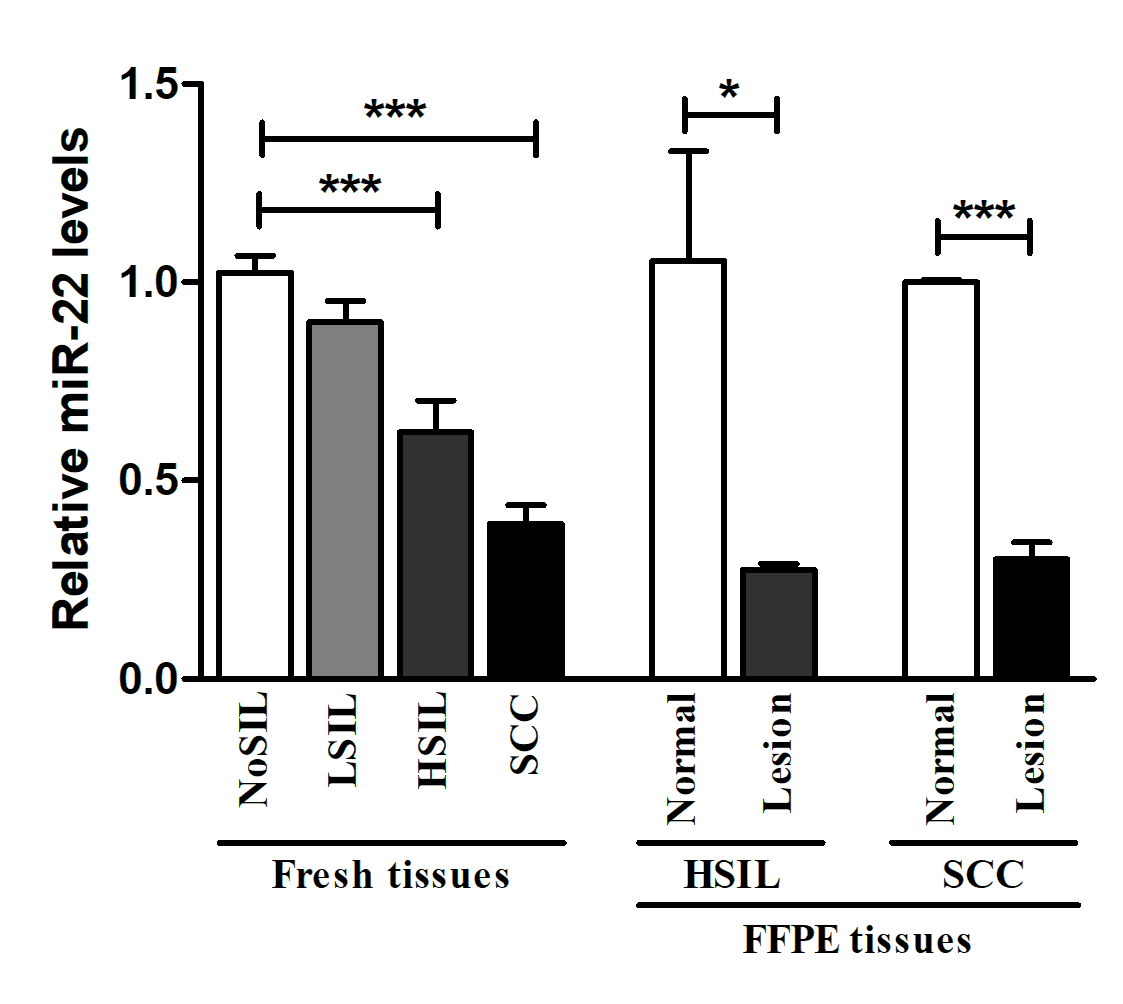
**

**Figure A. Comparison of miR-22 expression levels in fresh frozen and FFPE cervical cancer tissues.** The expression of miR-22 was examined by quantitative real-time reverse-transcriptase PCR (qRT-PCR) in fresh frozen cervical tissues including NoSIL, LSIL, HSIL and SCC and in FFPE tissues including HSIL and SCC. The levels of miR-22 were normalized with U44 small nuclear RNA. MiR-22 was down-regulated in HSIL and SCC samples compared with NoSIL samples but there was no difference between LSIL and NoSIL. In addition, the expression of this miRNA was also significantly lower in tumor regions compared with normal regions in both HSIL and SCC of FFPE samples. **P* < 0.05, ****P* < 0.001.

**
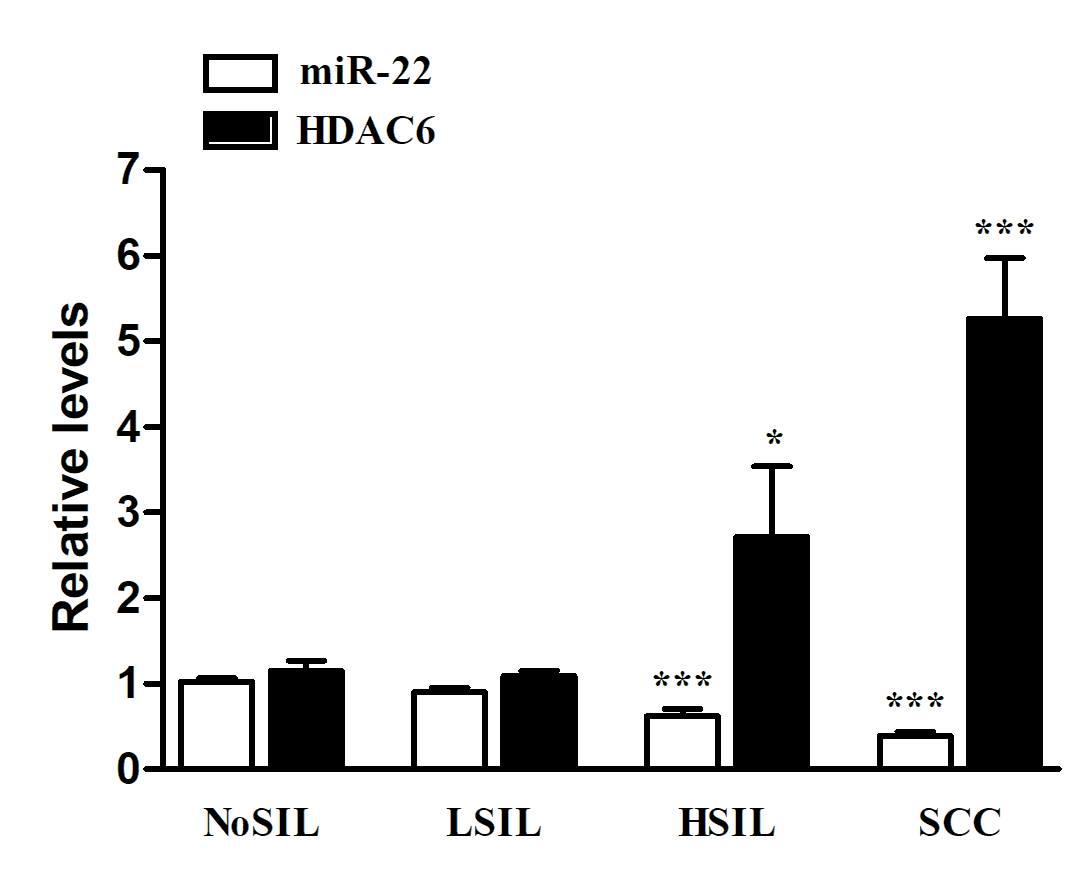
**

**Figure B.** **Comparison of miR-22 and HDAC6 mRNA expression levels in fresh frozen cervical cancer tissues.** The expression of miR-22 and HDAC6 were examined by quantitative real-time reverse-transcriptase PCR (qRT-PCR) in fresh frozen cervical tissues including NoSIL, LSIL, HSIL and SCC. The levels of miR-22 and HDAC6 were normalized with U44 small nuclear RNA and GAPDH, respectively. MiR-22 was down-regulated in HSIL and SCC samples compared with NoSIL samples but there was no difference between LSIL and NoSIL. In contrast, HDAC6 mRNA was significantly up-regulated in HSIL and SCC samples compared with NoSIL samples but there was no difference between LSIL and NoSIL. **P* < 0.05, ****P* < 0.001.

**Table A. Patient Characteristics.**

Clinical samples were collected from 2007 to 2011 (N=127).

| Characteristics | Number of cases |
| --- | --- |
| Age (years) |  |
| Mean ± SD | 45.6 ± 9.8 |
| Median (range) | 46.0 (23.0-78.0) |
|  |  |
|  | N (%) |
| Age group |  |
| < 50 | 80 (63.0%) |
| ≥ 50 | 47 (37.0%) |
| Histological grade |  |
| NoSIL | 30 (23.6%) |
| LSIL | 22 (17.3%) |
| HSIL | 31 (24.4%) |
| SCC | 44 (34.6%) |
| HPV status |  |
| HPV positive | 97 (76.4%) |
| HPV negative | 30 (23.6%) |

**Table B. DNA oligos sequences of miR-22 and HDAC6 3’UTR.**

| Name | DNA oligos sequences (5’-3’) | Sizes (bp) |
| --- | --- | --- |
| Pre-miR-22 | Sense:  AGGAGCCTGTTCCTCTCACGCCCTCACCTGGCTGAGCCGCAGTAGTTCTTCAGTGGCAAGCTTTATGTCCTGACCCAGCTAAAGCTGCCAGTTGAAGAACTGTTGCCCTCTGCCCCTGGCTTCGAGGAGGAGGAGGAGCTGCTTTCCCCATCATCTGGAAGGTGACAGAAATGGGCTGGGAAGGTCCGAACAGCAGGGTGGATGATACGTT | 211 |
|  | Antisense:  AACGTATCATCCACCCTGCTGTTCGGACCTTCCCAGCCCATTTCTGTCACCTTCCAGATGATGGGGAAAGCAGCTCCTCCTCCTCCTCGAAGCCAGGGGCAGAGGGCAACAGTTCTTCAACTGGCAGCTTTAGCTGGGTCAGGACATAAAGCTTGCCACTGAAGAACTACTGCGGCTCAGCCAGGTGAGGGCGTGAGAGGAACAGGCTCCT | 211 |
| HDAC6 3’UTR | Sense:  GCTGTAGCTCATTCCAGCCTGTACCTTGGATGAGGGGTAGCCTCCCACTGCATCCCATCCTGAATATCCTTTGCAACTCCCCAAGAGTGCTTATTTAAGTGTTAATACTTTTAAGAGAACTGCGACGATTAATTGTGGATCTCCCCCTGCCCATTGCCTGCTTGAGGGGCACCACTACTCCAGCCCAGAAGGAAAGGGGGGCAGCTCAGTGGCCCCAAGAGGGAGCTGATATCATGAGGATAACATTGGCGGGAGGGGAGTTAACTGGCAGGCATG | 276 |
|  | Antisense:  CATGCCTGCCAGTTAACTCCCCTCCCGCCAATGTTATCCTCATGATATCAGCTCCCTCTTGGGGCCACTGAGCTGCCCCCCTTTCCTTCTGGGCTGGAGTAGTGGTGCCCCTCAAGCAGGCAATGGGCAGGGGGAGATCCACAATTAATCGTCGCAGTTCTCTTAAAAGTATTAACACTTAAATAAGCACTCTTGGGGAGTTGCAAAGGATATTCAGGATGGGATGCAGTGGGAGGCTACCCCTCATCCAAGGTACAGGCTGGAATGAGCTACAGC | 276 |

**Table C. Nucleotide sequences of specific primers.**

| Targets | Primer sequences (5’ to 3’) | Product sizes (bp) |
| --- | --- | --- |
| HPV E6 | F: GTTACTGCGACGTGAGGTATATG | 90 |
|  | R: CATTTATCACATACAGCATATGGATTC |  |
| HDAC6 | F: TGGCTATTGCATGTTCAACCA | 127 |
|  | R: GTCGAAGGTGAACTGTGTTCCT |  |
| GAPDH | F: TCATCAGCAATGCCTCCTGCA | 117 |
|  | R: TGGGTGGCAGTGATGGCA |  |
| Pre-miR-22 | F: GGTACCAGGAGCCTGTTCCTCTCACGCC | 223 |
|  | R: GGATCCAACGTATCATCCACCCTGCTGT |  |
| HDAC6 3’-UTR | F: ATCTCGAGGCTGTAGCTCATTCCAGCCT | 294 |
|  | R: ATGCGGCCGCCATGCCTGCCAGTTAACT |  |

**Table D. PCR conditions.**

| Primer | PCR condition |
| --- | --- |
| Pre-miR-22 | Initial denaturation at 95^°^C for 5 minutes, followed by 40 cycles of 95^°^C for 30 seconds, 60^°^C for 45 seconds and 72^°^C for 1 minute. |
| HDAC6 3’-UTR | Initial denaturation at 95^°^C for 5 minutes, followed by 40 cycles of 95^°^C for 30 seconds, 65^°^C for 45 seconds and 72^°^C for 1 minute. |

**Table E. Real-time PCR conditions.**

| Primer | Real-time PCR condition |
| --- | --- |
| HPV E6 | Initial denaturation at 95^°^C for 2 minutes, followed by 40 cycles of 95^°^C for 5 seconds and 60^°^C for 1 minute. |
| p53 | Initial denaturation at 95^°^C for 2 minutes, followed by 40 cycles of 95^°^C for 5 seconds and 60^°^C for 1 minute. |
| HDAC6 | Initial denaturation at 95^°^C for 2 minutes, followed by 40 cycles of 95^°^C for 5 seconds and 60^°^C for 1 minute. |
| GAPDH | Initial denaturation at 95^°^C for 2 minutes, followed by 40 cycles of 95^°^C for 5 seconds and 60^°^C for 1 minute. |
